# Prostaglandin-E_2_ is produced by adult human epidermal melanocytes in response to UVB in a melanogenesis-independent manner

**DOI:** 10.1111/j.1755-148X.2010.00696.x

**Published:** 2010-03-17

**Authors:** Karl Gledhill, Lesley E Rhodes, Margaret Brownrigg, Ann K Haylett, Mojgan Masoodi, Anthony J Thody, Anna Nicolaou, Desmond J Tobin

**Affiliations:** 1 Centre for Skin Sciences, School of Life Sciences, University of BradfordBradford, UK; 2 Photobiology Unit, Dermatological Sciences, Manchester Academic Health Science Centre, University of Manchester, Salford Royal NHS Foundation HospitalManchester, UK; 3 School of Pharmacy, School of Life Sciences, University of BradfordBradford, UK; 4 School of Clinical and Laboratory Sciences, University of NewcastleNewcastle, UK

**Keywords:** epidermal melanocyte, ultraviolet radiation, prostaglandin E_2_, cyclooxygenase, melanogenesis, skin phototype

## Abstract

Excessive ultraviolet radiation (UVR) exposure induces erythema, mediated in part by prostaglandin-E_2_ (PGE_2_). While keratinocytes are a major PGE_2_ source, epidermal melanocytes (EM) also express PGE_2_-production machinery. It is unclear whether EM-produced PGE_2_ contributes to UVR-induced skin inflammation, and whether this is correlated with melanogenesis. Epidermal melanocytes were cultured from skin phototype-1 and -4 donors, followed by assessment of PGE_2_ production and melanogenesis. Epidermal melanocytes expressed cytoplasmic phospholipase-A_2_, cyclooxygenase-1, cytoplasmic prostaglandin-E synthase and microsomal prostaglandin-E synthase-1, -2. Epidermal melanocytes produced PGE_2_ under basal conditions, which increased further after arachidonic acid stimulation. Epidermal melanocytes expressed cyclooxygenase-2 (COX-2) mRNA and a selective COX-2 inhibitor (NS-398) reduced PGE_2_ production. Ultraviolet B-induced PGE_2_ production was positively correlated with skin phototype-1, despite variability between individual EM donors. By contrast, there was no correlation between PGE_2_ production by EM and their melanogenic status. Thus, EM may contribute to UVR-induced erythema, with role of donor skin phototype more important than their melanogenic status.

**Significance**

Low skin phototype individuals fail to tan effectively and are also more likely than higher skin phototypes to experience a PGE_2_-associated erythema after excessive UVR-exposure. The nature of this inverse relationship between melanogenesis and UVR-induced erythema remains unclear. Here, we show that UVR can stimulate PGE_2_ synthesis in normal human epidermal melanocytes in primary culture and remarkably does so in a skin phototype-dependent but melanogenesis-independent manner. Thus, skin phototype may be a better predictor than absolute melanin content of how melanocytes may contribute to the UVR-associated inflammatory cascade in human skin.

## Introduction

Exposure of intact skin to ultraviolet radiation (UVR) results in a number of responses including melanogenesis, generation of oxidative stress, vasodilation (skin erythema) and leukocyte infiltration (Tran et al., 2008). Vasodilation is known to be mediated in part by the pro-inflammatory eicosanoid prostaglandin-E_2_ (PGE_2_), with nitric oxide (NO) also contributing (Rhodes et al., 2001, 2009). Prostaglandin-E_2_ is present in blister fluid obtained from ultraviolet B (UVB)-irradiated skin exhibiting erythema, while treatment with indomethacin [a cyclooxygenase (COX) inhibitor] immediately after UVB irradiation reduces the erythema (Rhodes et al., 2001, 2009). Similarly, inhibition of NO synthases with *L*-NAME also reduces UVB-erythema (Rhodes et al., 2001). A major source of PGE_2_ and NO is thought to be the keratinocyte, as these cells produce these inflammatory mediators in response to UVB (Kang-Rotondo et al., 1993; Roméro-Graillet et al., 1997). However, more recently it has been suggested that epidermal melanocytes (EM) may also be a source of these inflammatory mediators, as these cells express some of the cellular machinery required for PGE_2_ production, i.e. COX-1 and COX-2 mRNA (Nicolaou et al., 2004). Moreover, EM have also been shown to produce NO in greater amounts than keratinocytes (Tsatmali et al., 2000).

Ultraviolet radiation (both UVA and UVB) is known to increase levels of oxidative stress within cells (Malorni et al., 1996), which can activate cytoplasmic phospholipase A_2_ (cPLA_2_) (Chen et al., 1996). Cytoplasmic phospholipase A_2_ hydrolyses arachidonic acid (AA) from membrane phospholipids, thus rendering it as a substrate for eicosanoid formation (Burke and Dennis, 2009). Free AA can be converted enzymatically to prostaglandin H_2_ (PGH_2_) by the action of the COX enzymes and then to PGE_2_ by the action of the terminal prostaglandin E synthase (PGES) enzymes (Murakami et al., 2002). Three isoforms of PGES have been described; two with preferential coupling to the two COX pathways (cPGES couples preferentially to COX-1 and mPGES-1 to COX-2, while mPGES-2 shows little preference for either pathway) (Murakami et al., 2003).

Ultraviolet radiation-induced erythema is reported to be greater in the skin of individuals who contain less melanin [i.e. skin phototype-1 and 2 (SPT-1 and 2)], than in more pigmented skin phototypes (SPT 3–6) (Li and Chu, 2007). Therefore, we have hypothesized that EM that do not or cannot produce significant melanin may be more likely instead to produce PGE_2_. In this way, these EM may contribute more to inflammation than to melanin production. However, it is not completely understood whether EM in the skin of individuals with SPT-1 and 2 are intrinsically incapable of producing melanin or simply not receiving relevant paracrine cues from their keratinocyte partner in the epidermal–melanin unit (Fitzpatrick and Breathnach, 1963). Therefore, it would be of significant interest to determine whether there is a correlation between PGE_2_ production by EM in response to UVR and their capacity for melanogenesis, as this could suggest that melanocytes in low or ‘non-tanning’ individuals may be more susceptible to contributing to UVR-induced inflammation. The expression and activity of melanogenic enzymes [including tyrosinase, tyrosinase-related protein-1 (TRP-1) and dopachrome tautomerase (DCT)] are important in determining the amount and type of melanin produced by EM. For example, whether eumelanin is composed of 5,6-dihydroxyindole (brown) or 5,6-dihydroxyindole carboxylic acid (black) subunits depends on the expression and activity of DCT (Maeda et al., 1997).

The aim of this study was threefold. First, we investigated whether EM can actually produce PGE_2_ under baseline (or AA-stimulated) conditions and whether this production is altered after UVR-exposure. Second, we assessed which components of the PGE_2_ synthetic pathway are expressed by normal human adult primary EM, and so attempted to determine the likely route for PGE_2_ production in these cells. Third, we investigated whether there is a correlation between PGE_2_ production by EM in response to UVB and their capacity for melanogenesis, so that we may provide evidence as to how PGE_2_ production may be regulated in these cells.

Results of this study showed that EM can indeed produce PGE_2_ under a number of experimental conditions, including in response to UVB. There may be multiple potential enzymatic routes to PGE_2_ formation by EM which may highlight the importance of this pro-inflammatory eicosanoid to EM. The level of production of this pro-inflammatory mediator was variable between primary cultures of different EM donors, but appeared to correlate overall with donor skin phototype. By contrast, PGE_2_ production did not correlate with baseline melanin levels, tyrosinase expression/activity or TRP-1 or DCT expression. Therefore, we suggest that an individual’s skin phototype may be a more useful indicator of their EM ability to produce PGE_2_ and that melanogenesis and PGE_2_ production appear to be unrelated in EM.

## Results

### EM produce PGE_2_ under both basal and AA-stimulated conditions

**Figure 1:**
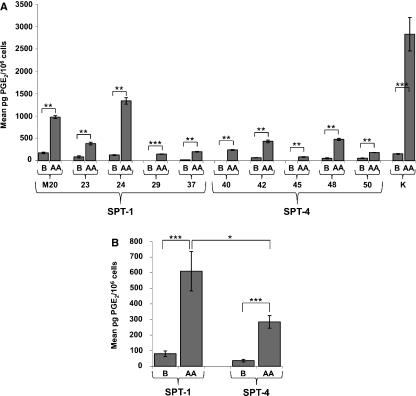
Epidermal melanocytes (EM) and keratinocytes (K) produce prostaglandin-E_2_ (PGE_2_) under basal and arachidonic acid (AA) stimulated conditions. (A) AA (3 μM) incubation for 24 h increased PGE_2_ production from basal levels in all 10 primary EM cultures and 1 primary K culture. (B) EM data shown in figure (A) pooled by skin phototype. B (basal conditions), AA (3 μM AA for 24 h), SPT (skin phototype). Cell cultures: M20, 23, 24, 29, 37, 40, 42, 45, 48, 50 and K. Error bars expressed as mean ± SEM: (A) n = 3, (B) n = 15. P < 0.01 = **, P < 0.001 = ***.

The levels of PGE_2_ production by EM were low under basal conditions (Figure 1A). Seven of 10 donor-derived primary EM cultures produced detectable amounts of PGE_2_ under basal conditions (Figure 1A). Levels of basal PGE_2_ production in these cells ranged from 20 (±1) pg/million cells to 176 (±15) pg/million cells. In order to investigate the intrinsic potential of EM for PGE_2_ production exogenous AA was added to the culture medium. After stimulation with exogenous AA (3 μM for 24 h), PGE_2_ production was markedly increased and was now detectable in all 10 donor-derived primary EM cultures investigated (Figure 1A), confirming the presence of active COX and PGES isoforms in these cells. Furthermore, the level of PGE_2_ production by these AA-stimulated primary EM cultures was again highly variable, ranging from 86 (±8) pg/million cells to 1339 (±73) pg/million cells. Keratinocytes (used as a positive control for PGE_2_ production) produced PGE_2_ at a level of 155 (±9) pg/million cells under basal conditions, and significantly more, 2829 (±373) pg/million cells, after AA stimulation.

### Production of PGE_2_ in response to AA is greater in SPT-1-derived EM than SPT-4-derived EM

In order to investigate the effect of skin phototype of the EM donor on the potential for PGE_2_ production in response to AA, data from all SPT-1 and SPT-4 EM cultures were pooled by skin phototype (Figure 1B). Data showed that EM from SPT-1 donors produced on average more PGE_2_ than EM derived from individuals with SPT-4 in response to AA (i.e. SPT-1 EM produced PGE_2_ at a level of 610 (±126) pg/million cells post-AA stimulation, while EM from SPT-4 donors produced PGE_2_ at 285 (±40) pg/million cells post-AA stimulation.

### EM express the cellular machinery required to produce PGE_2_

**Figure 2:**
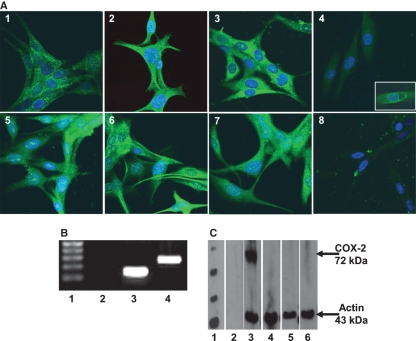
Epidermal melanocytes (EM) under basal conditions express the cellular machinery required to produce prostaglandin-E_2_ (PGE_2_). (A) Immunocytochemical detection of PGE_2_ enzyme machinery in EM. (A1) gp-100 protein (positive control for EM), (A2) cPLA_2_ protein, (A3) cyclooxygenase-1 (COX-1) protein, (A4) very weak or undetectable COX-2 protein in EM, inset positive control for COX-2 in keratinocytes, (A5) cPGES protein, (A6) mPGES-1 protein, (A7) mPGES-2 protein, (A8) negative control (no primary antibody). (B) PCR of COX-2 enzyme in EM. (B1) molecular weight marker, (B2) negative control (no cDNA), (B3) actin mRNA, (B4) COX-2 mRNA. (C) Western Blotting analysis of COX-2 enzyme in EM. (C1) molecular weight marker, (C2) negative control (no primary antibodies), (C3) actin and COX-2 protein in FM3 melanoma cells (COX-2 positive control), (C4) actin protein but lack of COX-2 protein expression under basal conditions, (C5) actin protein but lack of COX-2 protein expression 24 h post 73 mJ/cm^2^ ultraviolet B (UVB), (C6) actin protein but lack of COX-2 protein expression 48 h post 50 μM H_2_O_2_. EM used in figure (A) was derived from individual 37 skin phototype-1 (SPT-1) but is representative of results obtained from 10 different primary EM cultures. EM used in figures (B) and (C) were derived from individual 37 but is representative of results obtained from three different primary EM cultures (i.e. 23, 37 and 48). All lanes are from the same blot (i.e. experiment) and so are directly comparable.

The expression of enzymes required for the production of PGE_2_ (Murakami et al., 2002) was investigated in EM. Primary EM cultures from all 10 donors expressed cPLA_2_, COX-1, cPGES, mPGES-1 and mPGES-2 proteins (Figure 2A). By contrast, COX-2 protein could not clearly be detected in EM cultures from any of the 10 donors either under basal conditions, in response to UVB irradiation (73 mJ/cm^2^) or after exposure to H_2_O_2_ (50 μM for 48 h) (Figure 2C). However, COX-2 mRNA was detected in these cells (Figure 2B).

### Indomethacin and NS-398 reduce PGE_2_ production in EM

**Figure 3:**
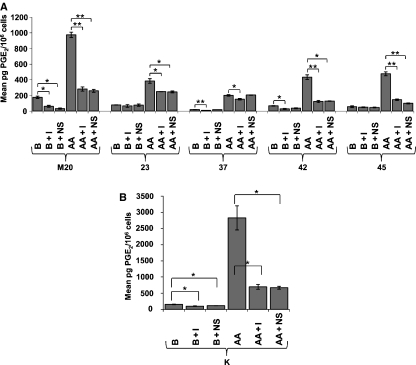
Indomethacin and NS-398 reduce prostaglandin E_2_ (PGE_2_) production in epidermal melanocytes (EM) and keratinocytes (K). (A) Incubation of EM with indomethacin or NS-398 reduced PGE_2_ production from basal or arachidonic acid (AA) stimulated levels in five primary EM cultures and (B) a primary keratinocyte culture. B (basal conditions), B + I (100 μM indomethacin for 1 h prior to 24 h stimulation period), B + NS (50 μM NS-398 for 1 h prior to 24 h stimulation period), AA (3 μM AA for 24 h), AA + I (100 μM indomethacin for 1 h prior to stimulation with AA and for 24 h during stimulation), AA + NS (50 μM NS-398 for 1 h prior to stimulation with AA and for 24 h during stimulation). Cell cultures: M20, 23, 37, 42, 48 and K. Error bars expressed as mean ± SEM: (A) n = 3, (B) n = 15. P < 0.05 = *, P < 0.01 = **.

In order to further investigate the involvement of the two COX isoforms in PGE_2_ production in EM, indomethacin (a non-selective COX inhibitor) and NS-398 (a selective COX-2 inhibitor) were employed. In four out of five EM cultures investigated, both indomethacin and NS-398 reduced PGE_2_ production. For example, under basal conditions indomethacin reduced PGE_2_ production in M20 EM from 176 (±15) pg/million cells to 61 (±13) pg/million cells and NS-398 reduced PGE_2_ production to 31 (±8) pg/million cells (Figure 3A). However, in the primary EM culture obtained from individual 37, only indomethacin reduced PGE_2_ production i.e. from 20 (±1) pg/million cells under basal conditions to 9 (±1) pg/million cells post-indomethacin stimulation (Figure 3A). Both indomethacin and NS-398 reduced PGE_2_ production in the keratinocyte primary culture. For example, under basal conditions indomethacin reduced PGE_2_ production from 155 (±9) pg/million cells to 100 (±7) pg/million cells and NS-398 reduced PGE_2_ production to 114 (±3) pg/million cells (Figure 3B).

### PGE_2_ production in EM is increased by UVB

**Figure 4:**
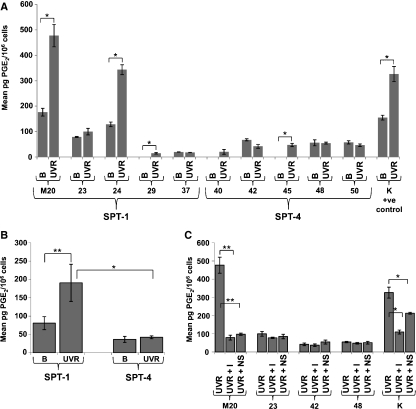
Prostaglandin E_2_ (PGE_2_) production in epidermal melanocytes (EM) and keratinocytes (K) is modulated by 73 mJ/cm^2^ ultraviolet B (UVB). (A) UVB increased PGE_2_ production in 6 of 10 primary EM cultures (4 at statistical significance) and 1 keratinocyte culture but had no effect in 4 of 10 EM cultures. (B) PGE_2_ production in response to UVB is increased significantly only in EM derived from individuals with skin phototype-1 (SPT-1). (C) Incubation with both indomethacin and NS-398 reduced PGE_2_ production in response to UVB in 1 of 4 EM cultures tested and 1 keratinocyte culture tested. B (basal conditions), UVR (73 mJ/cm^2^ UVB), UVR + I (1 h preincubation with 100 μM indomethacin then irradiation with 73 mJ/cm^2^ UVB and then post-incubation with 100 μM indomethacin for 24 h), UVR + NS (1 h preincubation with 50 μM NS-398 then irradiation with 73 mJ/cm^2^ UVB and then post-incubation with 50 μM NS-398 for 24 h). Cell cultures: M20, 23, 42, 48 and K. In (A) and (C) error bars are expressed as mean ± SEM (n = 3), in (B) error bars are expressed as mean ± SEM (n = 15). P < 0.05 = *, P < 0.01 = **.

Ultraviolet B causes inflammation in the skin, in part mediated by PGE_2_ (Rhodes et al., 2001, 2009). In order to investigate the potential involvement of EM in the sunburn response, PGE_2_ production was examined in these cells in response to UVB. Prostaglandin E_2_ production was statistically and significantly increased in primary EM cultures from 4 of 10 donors (M20, 24, 29 and 45) with a positive trend in a further two cultures (23 and 40) 24 h after 73 mJ/cm^2^ UVB (Figure 4A). For example, in EM derived from individual M20, PGE_2_ production was increased from 176 (±15) pg/million cells under basal conditions to 479 (±44) pg/million cells post-UVB. The level of PGE_2_ synthesis remained unchanged in cells from four other individuals (37, 42, 48 and 50) (Figure 4A). Prostaglandin E_2_ production in keratinocytes was also increased in response to UVB, i.e. from 155 (±9) pg/million cells under basal conditions to 326 (±30) pg/million cells post-UVB (Figure 4C).

### Production of PGE_2_ in response to UVB is greater in SPT-1 EM than SPT-4 EM

In order to investigate the effect of skin phototype of EM donor on PGE_2_ production in response to UVB, data for all SPT-1 and SPT-4 EM cultures were pooled separately (Figure 4B). Only EM from SPT-1 donors responded to UVB with a statistically significant increase in PGE_2_ production [i.e. from an average of 81 (±18) pg/million cells under basal conditions to an average of 191 (±51) pg/million cells post-UVB].

### UVB-induced PGE_2_ production does not correlate with melanin content, tyrosinase expression/activity or TRP-1 or DCT expression in EM

**Figure 5:**
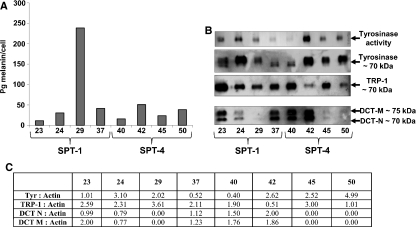
Basal melanin levels and melanogenic enzyme expression/activity in primary epidermal melanocyte (EM) cultures. (A) Basal melanin levels in primary EM cultures, (B) basal dopa-oxidase activity of tyrosinase and expression of tyrosinase, tyrosinase-related protein-1 (TRP-1) and dopachrome tautomerase (DCT) in primary EM cultures, (C) densitometric analysis of tyrosinase, TRP-1 and DCT expression normalized to actin expression. SPT (skin phototype). All lanes are from the same blot (i.e. experiment) and so are directly comparable. Cell cultures: 23, 24, 29, 37, 40, 42, 45 and 50. M = modified by glycosylation; N = native.

In order to investigate the hypothesis that EM in non-tanning skin may contribute to inflammation rather than melanogenesis, the levels of melanin production and melanogenic enzyme expression/activity were measured in primary EM cultures derived from eight donors. No correlation was observed with baseline melanin content, baseline expression/activity of tyrosinase or baseline expression of TRP-1 or DCT (Figure 5).

### The pro-oxidant H_2_O_2_ stimulated PGE_2_ production in EM

**Figure 6:**
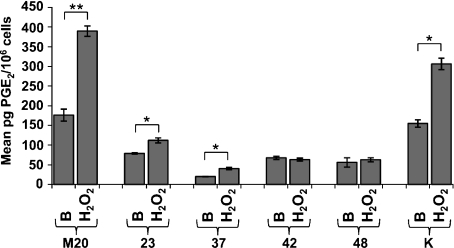
Epidermal melanocytes (EM) and keratinocytes (K) produce prostaglandin E_2_ (PGE_2_) in response to H_2_O_2_. Hydrogen peroxide (50 μM for 24 h) increased PGE_2_ production in three of five primary EM cultures tested and in one keratinocyte culture tested. B (basal conditions), H_2_O_2_ (50 μM H_2_O_2_ for 24 h). Cell cultures: M20, 23, 37, 42, 48 and K. Error bars expressed as mean ± SEM (n = 3). P < 0.05 = *, P < 0.01 = **.

Ultraviolet B exposure can lead to the generation of oxidative stress in cells. To investigate whether this may contribute to PGE_2_ production, EM was stimulated with the pro-oxidant H_2_O_2_. Three of the five EM cultures investigated (M20, 23 and 37) increased their production of PGE_2_ in response to H_2_O_2_ (50 μM for 24 h) (Figure 6). For example, in EM derived from individual M20, H_2_O_2_ increased PGE_2_ production from 176 (±15) pg/million cells under basal conditions to 390 (±13) pg/million cells post-H_2_O_2_ exposure. Prostaglandin E_2_ production in keratinocytes was also increased in response to H_2_O_2,_ i.e. from 155 (±9) pg/million cells under basal conditions to 306 (±15) pg/million cells post-H_2_O_2_ exposure (Figure 6).

## Discussion

Erythema occurs in the skin in response to excessive UVB exposure and this is in part mediated by PGE_2_ (Rhodes et al., 2001, 2009). Although a major source of this pro-inflammatory mediator is the keratinocyte (Kang-Rotondo et al., 1993), recent evidence suggests that EM may be potential additional contributors (Nicolaou et al., 2004). This study supports this view by showing that EM can produce PGE_2_ under different conditions, including in response to UVB exposure. The current study provides evidence to suggest that although keratinocytes have a greater potential for PGE_2_ production (as shown by their response to 3 μM AA stimulation), some EM can produce similar amounts in response to a physiologically relevant stimulus, i.e. 73 mJ/cm^2^ UVB. However, the 36:1 viable keratinocyte:melanocyte ratio in the epidermal–melanin unit (Fitzpatrick and Breathnach, 1963) indicates that keratinocytes are likely to be the dominant contributor of PGE_2_ in UV-irradiated skin. On the basis of our current data EM may still contribute to the PGE_2_ pool in UV-irradiated skin, and the position of EM near the dermo–epidermal junction may result in a proportionally greater influence (relative to the amount of PGE_2_ produced) on the dermal vasculature in vivo.

Melanoma cells have previously been shown to produce PGE_2_ under basal conditions (Nicolaou et al., 2004), while iridial melanocytes have been shown to produce PGE_2_ in response to latanoprost acid (Bergh et al., 2002). However, the enzymatic pathway used for PGE_2_ production in these latter reports was not elucidated. Data from the current study suggest that EM have a number of ways in which they can produce PGE_2_. These cells can convert free AA to prostaglandin G_2_ (PGG_2_) and then to PGH_2_ via either of the two COX isoforms, and then convert PGH_2_ to PGE_2_ by utilizing any of the three PGES. The ability by which EM produce PGE_2_ via a number of enzymatic pathways highlights the importance of this eicosanoid to the EM. Moreover, it has long been known that PGE_2_ can stimulate melanogenesis (Imokawa and Motegi, 1993) as a paracrine factor (e.g. from keratinocytes), and that EM express some PGE_2_ receptor isoforms (Scott et al., 2004). The production of this eicosanoid by EM themselves additionally suggests an autocrine role for PGE_2_.

Cyclooxygenase-2 protein was very weak to detect by immunofluorescence in EM in the current study, which concurs with findings of Goulet et al. (2003) who were also unable to detect COX-2 protein in EM. This finding may reflect the apparent inducible nature of COX-2, compared with the more general ‘house-keeping’ role of COX-1 (Sharma and Sharma, 1997). However, the ability of NS-398 (a selective COX-2 inhibitor) to reduce PGE_2_ production in some EM cultures clearly suggests a role for this enzyme in this system. Moreover, the ability of NS-398 to reduce PGE_2_ production to levels equal to those obtained when using a non-selective COX inhibitor (indomethacin) may even indicate that COX-2 is the major route by which PGE_2_ is produced in these cells. This report may provide evidence to further support this claim as similar results were obtained with keratinocytes, which are known to express COX-2 (Flockhart et al., 2008) as also shown in the current study. However, it would seem that for some EM donors COX-2 may have little involvement in PGE_2_ production. In these cells COX-1 may be the likely enzyme by which free AA is converted to PGH_2_. For example, in donor 37 EM, indomethacin but not the COX-2 specific inhibitor NS-398, reduced PGE_2_ production. The clear expression of COX-2 mRNA but not its associated protein (undetectable in the current study) in normal adult human EM could be explained by: (i) although the gene is actively transcribed much of the mRNA may not persist long enough to undergo translation, (ii) that translation is inhibited in some way or (iii) that the COX-2 protein product is rapidly turned over. In support of the latter, Mbonye et al. (2006) found that the half-life of COX-2 is about 2 h whereas the half-life of COX-1 is greater than 12 h in mouse fibroblasts. This may explain why COX-2 protein was not found in EM 24 h post-UVB exposure in the current study. It has also been shown that translation of COX-2 mRNA requires the activity of the eIF-5A transcription factor (Parker and Gerner, 2002), the inhibition of which impairs murine melanoma growth (Jasiulionis et al., 2007) and that basal COX-2 expression in melanoma cells is associated with malignant transformation (Kuźbicki et al., 2006). It remains unclear however, why COX-2 protein was still not detectable in EM after the 48 h exposure to the pro-oxidant H_2_O_2_. In support of this finding, Giandomenicoa et al. (1997) showed that H_2_O_2_ is rapidly degraded in cell culture medium therefore; it may be that after 48 h incubation the level of H_2_O_2_ was no longer adequate to induce COX-2 expression in these cells.

The current study suggests that while both AA- and UVB-induced PGE_2_ production by EM may be related to skin phototype of the EM donor, it is not correlated with EM melanin content, tyrosinase expression/activity or TRP-1 or DCT expression in vitro. Interestingly, EM derived from individuals with pale ‘non-tanning’ SPT-1 skin were still able to produce significant amounts of eumelanin with time in culture and at levels that were similar to those found in EM derived from individuals with ‘tannable’ SPT-4 skin. Therefore, it appears unlikely that there was any differential contribution of melanin to filtering of UVR at the level of the EM. In any event, UVR access to the plasma membrane would be similar for all EM cultures (SPT-1 or -4), as melanin is stored cytoplasmically within the EM. Moreover, given the similar melanin levels in both SPT-1- or SPT-4-derived EM, it is unlikely that UVR access to internal membranes would differ significantly. Thus, the observed differences in the ability of EM from different individuals to up-regulate PGE_2_ production after UVR- or AA-stimulation, could rather reflect the cells’ intrinsic membrane biochemistry and/or their relative expression/activity of PGE_2_-producing cellular machinery. Taken together, we propose that PGE_2_ production in EM in response to UVB is unrelated to intrinsic melanogenesis capacity and that skin phototype is instead largely determined by the nature of the keratinocyte partner in the epidermal–melanin unit [providing that the EM has an intact melanocortin 1 receptor (MC1R)]. In support of this interpretation it should be noted that we provide SPT-1-and SPT-4-derived EM with similar culture conditions, which included several pro-melanogenic growth factors [e.g. endothelin-1 (ET-1), basic fibroblast growth factor (bFGF), etc.]. These may be variably secreted by keratinocytes in the epidermal–melanin units of different SPT skin in situ.

The component of the UVR response, which stimulates production of PGE_2_ in EM and keratinocytes, is likely, at least in part, to involve oxidative stress, as PGE_2_ production was increased upon incubation with the pro-oxidant H_2_O_2_. Therefore, antioxidant capacity of the cell, rather than melanogenesis status per se, may determine the PGE_2_ response to UVB. Moreover, the classification of skin phototype may more accurately describe an individual’s propensity to ‘burn’ rather than to ‘tan’.

In conclusion, we confirm that EM have the capacity to produce PGE_2_, and show for the first time that the production of this pro-inflammatory eicosanoid in normal human EM can be increased by UVB exposure. However, we show that this response does not appear to occur in a melanogenesis-dependent manner. We also show the multiple enzymatic routes by which PGE_2_ can be produced in EM and therefore, highlight the importance of this eicosanoid to melanocyte function. Finally, we provide evidence to suggest that some EM can produce levels of PGE_2_ that are equal to or greater than keratinocytes in response to a physiologically relevant stimulus and so give an idea as to their relative contribution in producing the PGE_2_ pool in UVR-exposed epidermis. Therefore, we highlight the potentially important role EM may play in generating a UVB-induced erythema.

## Materials and methods

### Cell culture

Epidermal melanocytes were isolated from blister roofs derived from 10 healthy white Caucasian individuals (6F, 4M, 20–39 yr, mean 24.8 yr) with skin phototype-1 (n = 5) or 4 (n = 5) (see Table 1). Skin phototype was assessed according to a modified Fitzpatrick classification, i.e. historically from the individual’s reported propensity to sunburn and suntan following first exposure in summer, where phototype 1 always burns and never tans, while phototype 4 rarely burns but tans with ease (Fitzpatrick, 1975). Epidermal melanocytes were cultured [after selective trypsinization of keratinocytes and after removal of contaminating fibroblasts with 150 μg/ml geneticin sulphate (G418) in 48 h cycles at the p0 to p1 stage of primary culture (Halaban and Alfano, 1984)] in a mixture of keratinocyte–serum free medium (PromoCell, Heidelberg, Germany) supplemented with bovine pituitary extract (BPE) (4 μg/ml), epidermal growth factor (EGF) (0.125 ng/ml), insulin (5 μg/ml), hydrocortisone (0.33 μg/ml), epinephrine (0.39 μg/ml), transferrin (10 μg/ml), penicillin (100 U/ml)/streptomycin (100 μg/ml) and *L*-Gln (2 mM) and Eagle’s minimal essential medium supplemented with FBS (2%), 1x concentrated non-essential amino acid mixture, penicillin (100 U/ml)/streptomycin (100 μg/ml), *L*-Gln (2 mM), bFGF (5 ng/ml) and ET-1 (5 ng/ml) as previously described (Kauser et al., 2004). Melanocyte identity was confirmed by gp-100 immunostaining of cell monolayers at passage 1. Any residual and contaminating keratinocytes were removed by a further round of selective trypsinization. Melanoma cells (FM3) (used as a positive control for COX-2 expression) were cultured in RPMI 1640 culture medium (Invitrogen, Paisley, UK) supplemented with FBS (10%), penicillin (100 U/ml)/streptomycin (100 μg/ml) and *L*-Gln (2 mM). Epidermal keratinocytes (used as a positive control for PGE_2_ production) were isolated from one healthy Caucasian individual (Table 1) and cultured in keratinocyte–serum free medium (supplemented as described previously). Cells were incubated at 37°C in a 5% CO_2_ atmosphere and medium was replenished every third day.

**Table 1:** Clinical data of cell donors

	Sex	Skin phototype/tanning response (low = 1; high = 4)	Age (yr)
Epidermal melanocyte donor code			
M20	Male	1	20
23	Female	1	32
24	Male	1	39
29	Female	1	29
37	Female	1	27
40	Male	4	31
42	Female	4	26
45	Male	4	26
48	Female	4	24
50	Female	4	33
Keratinocyte donor code			
K	Female	2/3	60

### Stimulation of primary EM cultures

#### AA with/without indomethacin or NS-398

Prior to stimulation the primary cells were incubated for 24 h in ‘starved’ culture media (i.e. no FBS, BPE, EGF, insulin, hydrocortisone, epinephrine and transferrin). AA (3 μM) with/without 100 μM indomethacin or 50 μM NS-398 (Sigma, Poole, UK) were then added to the cells for 24 h. Cells were also incubated in parallel in control ‘starved’ culture media only without 3 μM AA but containing 100 μM indomethacin or 50 μM NS-398. After the 24 h stimulation period the culture media was collected for analysis of PGE_2_ concentration. In cells stimulated with 3 μM AA or 73 mJ/cm^2^ UVB (see below), 100 μM indomethacin or 50 μM NS-398 was added 1 h prior to the stimulation so that COX enzymes were inhibited before exposure to the stimulus.

#### Ultraviolet radiation

Primary cell cultures cultured in ‘starved’ medium (i.e. retaining bFGF and ET-1 as essential growth factors for EM viability) were temporarily submerged in PBS and irradiated with 73 mJ/cm^2^ UVB, using as a UVR source, a fluorescent UVB lamp (Waldmann UV6; emission 290–400 nm, peak 313 nm; Herbert Waldmann GmbH, Villingen-Schwenningen, Germany). Thus, the radiation consisted of approximately 34% UVA and 66% UVB. This radiation dose was calibrated on the basis of generating a mild induction of cellular loss (10%) from irradiated monolayers, as it was important to induce stress-associated signalling in these cells. The PBS was removed immediately after irradiation and replaced with fresh ‘starved’ media. This culture medium was collected after 24 h for analysis of PGE_2_ concentration. Control cells were treated similarly but not irradiated.

### Immunofluorescence

Primary EM cultures were incubated in ‘starved’ culture medium for 24 h and fixed in ice cold methanol (Sigma) for 10 min before air drying and rehydration in PBS. The cells were blocked with 10% donkey serum for 90 min and washed with PBS before incubation with primary antibody [gp-100 (1:50) (Monosan, Uden, The Netherlands), cPLA_2_ (1:200) (Santa Cruz Biotechnology, CA, USA), COX-1 (1:50) (Santa Cruz Biotechnology), COX-2 (1:200) (Cayman Chemicals, Ann Arbor, MI, USA), cPGES (1:400) (Cayman Chemicals), mPGES-1 (1:400) (Cayman Chemicals), mPGES-2 (1:400) (Cayman Chemicals)] overnight at 4°C. Thereafter, the cells were washed and incubated with the secondary antibody [Alexa Fluor 488 donkey anti-goat (1:250), donkey anti-rabbit (1:100) or donkey anti-mouse (1:100) (Invitrogen, Paisley, UK)] for 1 h at RT. Slides were then washed with PBS, cover-slipped using mounting medium containing 4′,6-diamidino-2-phenylindole (Vector, Peterborough, UK) and photographed using a confocal microscope at 630× original magnification.

### Polymerase chain reaction

RNA was extracted from primary EM cultures using RNeasy isolation kit (Qiagen, West Sussex, UK) according to the manufacturer’s instructions and quantified in a spectrophotometer at 260 nm. cDNA synthesis was performed with 3 μg of total RNA using superscript III first-strand synthesis super mix (Qiagen) according to the manufacturer’s instructions. Cyclooxygenase-2 was amplified using the primer set (Sigma): 5′-TTCAAATGAGATTGTGGGAAAATTGCT-3′ (forward) (0.125 μM) and 5′-AGATCATCTCTGCCTGAGTATCTTT-3′ (reverse) (0.125 μM) (Shi et al., 2006). Actin was amplified using the primer set (Sigma): 5′-TCACCCACACTGTGCCCATCTACGA-3′ (forward) (0.125 μM) and 5′-CAGCGGAACCGCTCATTGCCAATGG-3′ (reverse) (0.125 μM). The cycling protocol for both genes was: 95°C for 10 min; 45 cycles at 95°C for 0 min, 60°C for 20 s, 72°C for 30 s; and a final extension at 72°C for 1 min. The omission of cDNA from the reaction mixture served as a negative control. After amplification, 15 μl of the reaction mixture was loaded onto a 1.5% agarose gel (Sigma) containing 1 μg/ml of ethidium bromide (Sigma) and electrophoresed. A 100–1000 bp DNA ladder (Invitrogen, Paisley, UK) was also loaded.

### Western immunoblot analysis

The presence of COX-2 protein was assessed by immunoblotting human EM and FM3 extracts under unstimulated and stimulated conditions. The latter included 24 h post-irradiation with 73 mJ/cm^2^ UVB and 48 h post-incubation with 50 μM H_2_O_2_. The basal expression of tyrosinase, TRP-1 and DCT was assessed in human EM extracts. Cells were lysed on ice using Laemmli’s buffer containing a protease inhibitor cocktail (Sigma) for 4 h. Protein concentration was measured using the modified Bradford Assay (Bio-Rad, Richmond, CA, USA), and 35 μg of protein per well were separated by SDS–8% PAGE under reducing conditions. Separated proteins were then electroblotted onto polyvinylidene difluoride membranes (PVDF) (Immobilon, Millipore, Bedford, MA, USA), and blocked for 2 h with 5% nonfat milk (Marvel Ltd, Merseyside, UK) in PBS. The membranes were immuno-probed overnight at 4°C with antibodies against COX-2 (1:75) (Cayman Chemicals), tyrosinase (1:100) (Santa Cruz Biotechnology), TRP-1 (1:1000) (Santa Cruz Biotechnology), DCT (1:100) (Santa Cruz Biotechnology) and actin (1:250) (Santa Cruz Biotechnology), followed by incubation for 2 h at RT with a horseradish peroxidase-conjugated donkey anti-sheep/goat IgG antibody (1:600) (Serotec Ltd, Kidlington, Oxford, UK) or a horseradish peroxidase-conjugated donkey anti-rabbit IgG antibody (1:1000) (GE Healthcare, Chalfont St Giles, Buckinghamshire, UK). The reactions were detected by the Enhanced Chemiluminescence plus Western blot detection system kit (Amersham Biosciences Ltd, Little Chalfont, Buckinghamshire, UK). Densitometric analysis of the immunoblots was performed (TotalLab v1.10 software, Auckland, New Zealand) and the ratio of tyrosinase:actin, TRP-1:actin and DCT:actin expression levels calculated. This allowed for direct comparisons of expression of the aforementioned enzymes across different primary EM cultures. To reprobe the blots the PVDF membrane was incubated at 50°C for 30 min in a buffer containing 67.5 mM Tris–HCl, pH 6.8, 100 mM β-mercaptoethanol, and 2% SDS.

### Tyrosinase activity by DOPA-oxidase assay

Proteins were separated by SDS-PAGE under non-reducing conditions (i.e. without boiling and without the addition of β-mercaptoethanol). The PVDF membrane was incubated for 3 h in 10 mM 3, 4-dihydroxy-*L*-Phe (*L*-DOPA) (Sigma). Tyrosinase catalysed the conversion of *L*-DOPA to DOPA-quinone and produced a dark band on the membrane where the enzyme was localized.

### Melanin assay

Epidermal melanocyte pellets were re-suspended in 600 μl of 1 M sodium hydroxide solution and boiled at 100°C for 15 min. The cell suspension was vortexed and 200 μl of each EM sample was added (in triplicate) to a 96-well plate. Eumelanin concentration of each EM sample was calculated using a standard curve created with synthetic eumelanin (Sigma, Poole, UK) at 100, 50, 20, 10, 5 and 1 μg/ml with absorbance measured at 490 nm (Kauser et al., 2004).

### Prostaglandin E_2_ ELISA

The concentration of PGE_2_ in cell culture media was determined in triplicate using a commercially available enzyme-based immunoassay kit (Cayman Chemicals) in accordance with the manufacturer’s instructions. This assay kit can reliably quantify levels of PGE_2_ of 15 pg/ml, but can detect levels as low as 7 pg/ml of PGE_2_. The PGE_2_ antibody exhibits very high selectivity for PGE (100% with PGE_2_, 100% with PGE_2_ Ethanolamide, 43% with PGE_3_, 37.4% with 8-iso PGE_2_, 18.7% with PGE_1_) and only very minor cross-reactivity with other prostaglandins (e.g. 1.25% with sulprostone, 1.0% with 6-keto PGF_1α_, 0.25% with 8-iso PGF_2α_ and 0.02% with 13, 14-dihydro-15-keto PGE_2_) (Cayman Chemicals). All cell culture media used in this ELISA method was ‘starved’ as FBS may contain PGE_2_. This meant that any PGE_2_ that was detected was only produced by the cells being investigated.

### Statistical analysis

Data were analysed using Student’s t-test (two-tailed, type-1). Statistical significance was accepted at the P < 0.05 level (*), P < 0.01 level (**) and P < 0.001 level (***).

## References

[b1] Bergh K, Wentzel P, Stjernschantz J (2002). Production of prostaglandin E_2_ by iridial melanocytes exposed to latanoprost acid, a prostaglandin F_2α_ analogue. J. Ocul. Pharmacol. Ther..

[b2] Burke JE, Dennis EA (2009). Phospholipase A_2_ structure/function, mechanism, and signaling. J. Lipid Res..

[b3] Chen X, Gresham A, Morrison A, Pentland AP (1996). Oxidative stress mediates synthesis of cytosolic phospholipase A_2_ after UVB injury. Biochim. Biophys. Acta.

[b4] Fitzpatrick TB (1975). Soleil et peau. J. Med. Esthet..

[b5] Fitzpatrick TB, Breathnach AS (1963). The epidermal melanin unit system. Dermatol. Wochenschr..

[b6] Flockhart RJ, Diffey BL, Farr PM, Lloyd J, Reynolds NJ (2008). NFAT regulates induction of COX-2 and apoptosis of keratinocytes in response to ultraviolet radiation exposure. FASEB J..

[b7] Giandomenicoa AR, Cernigliaa CE, Biaglowa JE, Stevensa CW, Kocha CJ (1997). The importance of sodium pyruvate in assessing damage produced by hydrogen peroxide. Free Radic. Biol. Med..

[b8] Goulet AC, Einsphar JG, Alberts DS, Beas A, Burk C, Bhattacharyya A, Bangert J, Harmon JM, Fujiwara H, Koki A, Nelson MA (2003). Analysis of cyclooxygenase 2 (COX-2) expression during malignant melanoma progression. Cancer Biol. Ther..

[b9] Halaban R, Alfano FD (1984). Selective elimination of fibroblasts from cultures of normal human melanocytes. In Vitro.

[b10] Imokawa G, Motegi I (1993). Skin organ culture model for examining epidermal melanization. J. Invest. Dermatol..

[b11] Jasiulionis MG, Luchessi AD, Moreira AG, Souza PP, Suenaga AP, Correa M (2007). Inhibition of eukaryotic translation initiation factor 5A (eIF5A) hypusination impairs melanoma growth. Cell Biochem. Funct..

[b12] Kang-Rotondo CH, Miller CC, Morrison AR, Pentland AP (1993). Enhanced keratinocyte prostaglandin synthesis after UV injury is due to increased phospholipase activity. Am. J. Physiol..

[b13] Kauser S, Thody AJ, Schallreuter KU, Gummer CL, Tobin DJ (2004). Beta-endorphin as a regulator of human hair follicle melanocyte biology. J. Invest. Dermatol..

[b14] Kuźbicki L, Sarnecka A, Chwirot BW (2006). Expression of cyclooxygenase-2 in benign naevi and during human cutaneous melanoma progression. Melanoma Res..

[b15] Li YW, Chu CY (2007). The minimal erythema dose of broadband ultraviolet B in Taiwanese. J. Formos. Med. Assoc..

[b16] Maeda K, Yokokawa Y, Hatao M, Naganuma M, Tomita Y (1997). Comparison of the melanogenesis in human black and light brown melanocytes. J. Dermatol. Sci..

[b17] Malorni W, Straface E, Donelli G, Giacomoni PU (1996). UV-induced cytoskeletal damage, surface blebbing and apoptosis are hindered by a-tocopherol in cultured human keratinocytes. Eur. J. Dermatol..

[b18] Mbonye UR, Wada M, Rieke CJ, Tang HY, Dewitt DL, Smith WL (2006). The 19-amino acid cassette of cyclooxygenase-2 mediates entry of the protein into the endoplasmic reticulum-associated degradation system. J. Biol. Chem..

[b19] Murakami M, Nakatani Y, Tanioka T, Kudo I (2002). Prostaglandin E synthase. Prostaglandins Other Lipid Mediat..

[b20] Murakami M, Nakashima K, Kamei D, Masuda S, Ishikawa Y, Ishii T, Ohmiya Y (2003). Cellular prostaglandin E_2_ production by membrane-bound prostaglandin E synthase-2 via both cyclooxygenases-1 and -2. J. Biol. Chem..

[b21] Nicolaou A, Estdale SE, Tsatmali M, Herrero DP, Thody AJ (2004). Prostaglandin production by melanocytic cells and the effect of alpha-melanocyte stimulating hormone. FEBS Lett..

[b22] Parker MT, Gerner EW (2002). Polyamine-mediated post-transcriptional regulation of COX-2. Biochimie.

[b23] Rhodes LE, Belgi G, Parslew R, McLoughlin L, Clough GF, Friedmann PS (2001). Ultraviolet-B-induced erythema is mediated by nitric oxide and prostaglandin E_2_ in combination. J. Invest. Dermatol..

[b24] Rhodes LE, Gledhill K, Masoodi M, Haylett AK, Brownrigg M, Thody AJ (2009). The sunburn response in human skin is characterized by sequential eicosanoid profiles that may mediate its early and late phases. FASEB J..

[b25] Roméro-Graillet C, Aberdam E, Clément M, Ortonne JP, Ballotti R (1997). Nitric oxide produced by ultraviolet-irradiated keratinocytes stimulates melanogenesis. J. Clin. Invest..

[b26] Scott G, Leopardi S, Printup S, Malhi N, Seiberg M, Lapoint R (2004). Proteinase-activated receptor-2 stimulates prostaglandin production in keratinocytes: analysis of prostaglandin receptors on human melanocytes and effects of PGE_2_ and PGF_2α_ on melanocyte dendricity. J. Invest. Dermatol..

[b27] Sharma S, Sharma SC (1997). An update on eicosanoids and inhibitors of cyclooxygenase enzyme systems. Indian J. Exp. Biol..

[b28] Shi Q, Vaillancourt F, Côté V, Fahmi H, Lavigne P, Afif H, Di Battista JA, Fernandes JC, Benderdour M (2006). Alterations of metabolic activity in human osteoarthritic osteoblasts by lipid peroxidation end product 4-hydroxynonenal. Arthritis Res. Ther..

[b29] Tran TT, Schulman J, Fisher DE (2008). UV and pigmentation: molecular mechanisms and social controversies. Pigment Cell Melanoma Res..

[b30] Tsatmali M, Graham A, Szatkowski D, Ancans J, Manning P, McNeil CJ (2000). Alpha-melanocyte-stimulating hormone modulates nitric oxide production in melanocytes. J. Invest. Dermatol..

